# Childhood Dietary Intake in Italy: The Epidemiological “MY FOOD DIARY” Survey

**DOI:** 10.3390/nu11051129

**Published:** 2019-05-21

**Authors:** Elvira Verduci, Giuseppe Banderali, Chiara Montanari, Roberto Berni Canani, Luigi Cimmino Caserta, Giovanni Corsello, Fabio Mosca, Ruggiero Piazzolla, Maria Rescigno, Luigi Terracciano, Ersilia Troiano, Marina Crosa, Claudio Maffeis, Ruggiero Francavilla

**Affiliations:** 1 Department of Pediatrics, San Paolo Hospital, University of Milan, 20142 Milan, Italy; giuseppe.banderali@unimi.it (G.B.); chiara.montanari@unimi.it (C.M.); marina.crosa@unimi.it (M.C.); 2 Department of Translational Medical Science, University of Naples “Federico II”, 80131 Naples, Italy; berni@unina.it; 3 CEINGE Advanced Biotechnologies, University of Naples “Federico II”, 80131 Naples, Italy; 4 Task Force for Microbiome Studies, University of Naples “Federico II”, 80131 Naples, Italy; 5 European Laboratory for the Investigation of Food-Induced Diseases (ELFID), University of Naples “Federico II”, 80131 Naples, Italy; 6 Direction Medical, KraftHeinz Italia Via Migliara 45, 04100 Latina, Italy; luigi.cimminocaserta@kraftheinz.com; 7 Department of Sciences for Health Promotion and Mother and Child Care “G. D’Alessandro”, University of Palermo, 90133 Palermo, Italy; giocors@alice.it; 8 NICU Fondazione IRCCS Ca’ Granda Ospedale Maggiore Policlinico, University of Milan, 20122 Milano, Italy; fabio.mosca@unimi.it; 9 Representatives of the Apulian Federazione Italiana Medici Pediatri (FIMP), Via S. Antonio 73, 70051 Barletta, Italy; piaroger@tin.it; 10 Mucosal Immunology and Microbiota Unit, Humanitas Clinical and Research Center—IRCCS, Via Manzoni 56, 20089 Rozzano, Italy; maria.rescigno@hunimed.eu; 11 Pediatric Primary Care, National Pediatric Health Care System, 20100 Milan, Italy; terrycom1957@gmail.com; 12 Italian Association of Dietitians, 00100 Rome, Italy; ersilia.troiano@gmail.com; 13 Pediatric Diabetes and Metabolic Disorders, Department of Surgery, Dentistry, Paediatrics and Gynaecology, University of Verona, 37129 Verona, Italy; claudio.maffeis@univr.it; 14 Interdisciplinary Department of Medicine–Pediatric Section, University of Bari Aldo Moro, 70121 Bari, Italy; ruggiero.francavilla@uniba.it; 15 Ospedale Pediatrico Giovanni XXIII, via Amendola 276, 70125 Bari, Italy

**Keywords:** epidemiology, descriptive observational study, survey, nutrient intake, dietary pattern, food record, obesity, health, infants, toddlers

## Abstract

Promoting a healthy lifestyle during the first years of life is a key strategy for controlling obesity risk in later life; having good-quality epidemiological data on eating habits of infants and toddlers can improve awareness and possibly the education given by pediatricians to parents and children. With this aim, we performed a survey about the dietary pattern of Italian children in early childhood. We described the intake of energy, macronutrients and fiber, minerals, and vitamins of 443 Italian children (range 6.4–131 months), through a three-day food record filled out by their parents and assessed by family pediatricians. The results were compared with the Italian Dietary Reference Values. The median protein intake, in g/kg per body weight, exceeded the average requirement in all age groups, and in the 12–36 month period, the intake as % of energy was outside the reference range (>15%). The majority of the children consumed quantities of simple carbohydrates (consisting of both natural sugars and free or added sugars, 82.3% of the children in the study) and saturated fats (69% of the children in the study) above the limits of the Italian Dietary Reference Values, with low intake of fiber and polyunsaturated fats. Median mineral intake, in our study, was different depending on age, while vitamin D intake was very low in all age groups. This is one of the few studies reporting on the nutrient intake of Italian children with reference to nutrition recommendations in order to identify the principal nutritional errors. The present results underline the need for healthcare policies starting from the first years of life in order to ameliorate nutrient intake during childhood, possibly impacting long-term health outcomes.

## 1. Introduction

Consuming a healthy diet characterized by a variety of nutritious foods and a balanced intake of energy and macro- and micronutrients is essential for promoting and maintaining health and safety, especially throughout the early childhood years. Nutrition is one of the environmental factors, together with demographic, socioeconomic, behavioral, and motivational aspects, that affect the growth and development of the brain, on the foundation of genetic influence [1]. Nutrition plays an important role and since it is a modifiable factor, interventions to adjust incorrect eating habits in order to optimize the growth of children and cognitive development [2] have to be performed. Nutrition, particularly the interaction between micronutrients and macronutrients, seems to influence the development of the brain in terms of macrostructure (e.g., development of brain areas such as the hippocampus), microstructure (e.g., myelination of neurons), and level and operation of neurotransmitters (e.g., dopamine levels or receptor numbers), with an impact on cognitive development [2].

Obesity is defined as an excess of body fat [3] and is a public health problem [4] with a high and increasing prevalence [3,5]. Obesity at a pediatric age is increasing significantly, with a two- to threefold higher prevalence in the past few decades in the United States and in other countries. Because of the numerous medical and psychosocial complications related to childhood obesity and the influence on current and future health care costs, this condition is now recognized as a major public health problem by many experts [6]. Obesity affects several age groups [4], but represents a major health challenge for children and adolescents [5] due to the complexity of the treatment, the association with other non-transmissible diseases, and the high likelihood of persistence into adulthood [7]. Physical and psychosocial complications seem to be already present in obese children [4,8]. Several longitudinal studies highlight the strong association of pediatric obesity with the persistence of adult obesity; excess of adiposity in childhood is a marker of increased cardiometabolic risk in adolescents and adults, and risk of future development of cardiovascular disease and diabetes, as well as a reduced life expectancy [9]. Epidemiological and experimental studies suggest a relation between early dietary patterns and a later risk of obesity [5]. Childhood and adolescence obesity are caused by a chronic energy imbalance involving both dietary intake and physical activity [1]. A high consumption of energy-dense food, meal skipping, and a high intake of saturated fat, sugar, and salt, associated with high levels of sedentarism, are the main causes of obesity [7]. The aim is to achieve optimal cognitive development and adequate growth, lowering the risk of obesity and chronic disease through suitable eating and habits as well as regular physical activity [10]. The first years of life are pivotal to starting preventive interventions [1] and ameliorating nutrition can benefit growth as well as cognitive and behavioral development, possibly also preventing a later onset of obesity and complications. Analysis of dietary intakes may be very important for public health, mainly to define and optimize nutrition policies and guidelines. Additionally, monitoring population food consumption with reference to nutrition recommendations may help in identifying links between diet and disease [11].

Nowadays, there is still a worldwide need for epidemiological data describing and analyzing nutritional practices and dietary intake in early childhood, especially in preschool children, where this is more challenging since they consume their lunch at home [12]. The aim is to improve education and knowledge and provide guidance towards a healthy and modernized culture of nutrition, especially with respect to pediatricians, parents, and children.

A previous national study reported the nutrient intake in Italian infants and toddlers aged 6 to 36 months from north and south Italy [12]. No statistically significant differences were found in the median daily intake of energy and macronutrients between Milan and Catania, in accordance with a similar anthropometric status. Only the intake of iron was lower, and the intake of fiber was greater in Milan than in Catania in a biologically relevant way [12]. Furthermore, in comparison with the Italian Dietary Reference Values, there was a generally high intake of proteins, simple sugars, saturated fatty acids, and sodium, and a generally low intake of iron and fiber.

To our knowledge, no recent study has evaluated the macro- and micronutrient intake in a cohort of Italian children so numerous and with such a wide age range [12].

This survey primarily aimed to describe the dietary intake in terms of energy and macro- and micronutrients (calcium, sodium, iron, zinc, and vitamin D) in Italian children.

## 2. Materials and Methods 

### 2.1. Study Design 

This is a descriptive observational study conducted among children, their families, and the family pediatricians practicing in Italy and included in the registries of the local health authorities. 

The survey planned to collect data from a general clinic visit and anthropometric assessment performed by the family pediatrician, as well as nutrition data from parents who filled out three-day food records (including two weekdays and one weekend day). Parents were also taught about and trained regarding the methods for correctly weighing each food item offered to their child before consumption and filling in the food records. Each pediatrician was requested to support parents in filling in the three-day records whenever needed. All survey data on children and their families were transferred to the reference centre (Department of Pediatrics, San Paolo Hospital, Milan, Italy) for analysis. 

Institutes, the San Paolo Hospital, and family pediatricians involved in the survey called "My food diary", were authorized to perform research and clinical studies by the Ministry of Health. Authorized investigators contacted the pediatricians and those willing to participate in the study gave their oral informed consent. The pediatricians obtained informed consent to participate in the study from parents after providing a detailed explanation of the study, allowing their enrolment in non-active intervention clinical studies that guarantee anonymity. An informed privacy consent form was also signed by the parents. Thus, separate ethics approval for the present survey was not required as no patient’s personal information was collected, and the study design satisfied the criteria of an activity audit.

### 2.2. Recruitment Procedure

An Excel spreadsheet was sent to all registered *Federazione Italiana Medici Pediatri* (FIMP) members, inviting them to participate voluntarily.

The attending pediatricians had to ask three parents of “healthy” children aged 6–36 months and three parents of “healthy” children aged >36 months to participate in the study; participation was voluntary. Pediatricians had to ascertain whether the inclusion criteria for children were satisfied before recruiting. Inclusion criteria involved age (children aged 6–36 months or >36 months), ethnicity (Caucasian), and health status (no chronic disease or malformation, no chronic use of drugs). Recruitment was performed by pediatricians randomly, on two established days of the week (e.g., Monday and Tuesday) during outpatient visits.

Each pediatrician sent an anonymized list of recruited children, with a unique pediatrician identification code, a unique patient number, and the sex and birthdate of each patient.

### 2.3. Collection of Data from Family Pediatricians

The family pediatricians collected socio-demographic, nutritional, and anthropometric data of parents, including their age and self-reported weight and height. They also obtained retrospective data about the initiation of breastfeeding and the rate of breastfeeding at 180 days of life. The breastfeeding practices were in accordance with WHO, as updated in 2007 [13]. Breastfeeding was defined as the feeding practice allowing the infant to receive breast milk and allowing the infant to receive anything else. “Initiation” of breastfeeding was defined as breastfeeding which had started within 48 h post-delivery. 

The body mass index (BMI) was calculated and classified according to the World Health Organization (WHO) [14,15]. The family pediatricians performed anthropometric measurements of children, including weight (kg) and length/height (cm). Standard scores (*z*-scores) of weight, length/height, and BMI were subsequently calculated at the Department of Pediatrics, San Paolo Hospital, Milan, using WHO reference data for children [15] and the WHO Anthro (version 3.2.2, January 2011) software (Geneva, Switzerland) with macros (available from http://www.who.int/childgrowth/software/en/).

### 2.4. Collection and Assessment of Data for Dietary Intake

For each participating child, the dietary intake was evaluated by three-day food records filled out by parents, including three consecutive days (two weekdays and one weekend day). Parents received instructions and were educated by family pediatricians on the method for weighing and recording food. After compilation, parents returned the completed records to the referenced pediatricians, who, after checking the quality and completeness, and making data anonymous, sent the food records together with data he/she gathered during the general visit to the reference centre (Department of Pediatrics, San Paolo Hospital, Milan, Italy). A dietician entered all the data into fitted case report forms after creating a working food list of unique items on the basis of food name and brand. Quantification and analysis of the energy intake and nutrient composition were performed by using ad hoc PC software (MètaDieta_, Me.Te.Da S.r.l., San Benedetto del Tronto, Italy).

### 2.5. Statistical Analysis

Descriptive statistics for continuous variables are reported as the mean (standard deviation (SD)), median (i.e., 50th centile), 25th and 75th centiles, and minimum and maximum for both Gaussian and non-Gaussian distributed variables, to provide the reader with a complete description of data. Discrete variables are reported as the number of observations and percentage, as pertinent. Age groups were expressed as intervals of months and/or years, as appropriate, and categorized firstly in accordance with [16], to allow an immediate and truthful readability of the results with respect to the nutrition recommendations [16]. The Italian Dietary Reference Values give different nutrition reference values for the following age groups: 6–12 months (6 ≤ months < 12), 1–3 years (1 ≤ years < 4), 4–6 years (4 ≤ years < 7), and 7–10 years (7 ≤ years < 11) [16]. Therefore, we split the group aged 6–36 months into two subgroups, 6–12 months and 1–3 years (for <6-month-old infants no reference values are available), whilst children aged >36 months were differentiated in the 4–6 year and 7–10 year age subgroups; children >36 months but <4 years were included in the 1–3 year age group since they have the same reference values. No results from inferential analysis were reported in this document given its descriptive character, and more relevantly, to maintain neutrality and avoid any prejudice given the doubtful representativeness of the analyzed sample of the general Italian early childhood population. The SPSS statistical package, version 20.0 (SPSS Inc., Chicago, IL, USA) for Windows (Microsoft, Redmond, WA, USA), was used for the statistical analysis. 

### 2.6. Sample Size

The sample size was determined assuming a reference adherence rate based on the Italian Dietary Reference Values (age- and sex-adjusted reference intake for macronutrients) [16] of 71% [12]. For estimating the expected adherence with 5% absolute precision and 95% confidence, a sample of at least 317 participant children is required [17]. With an actual number of 443 children analyzed, the study was able to estimate the expected adherence with an absolute precision of approximately 4.2%, with a 95% confidence level.

## 3. Results

### 3.1. Characteristics of the Study Population

The study analyzed the three-day food records of 443 children (227 girls and 216 boys), enrolled by their family pediatricians. Eighty family pediatricians participated. Table 1 shows the regional distribution of participating family pediatricians and children; the most represented area is Area 4 (Abruzzo, Molise, Puglia, Campania, Basilicata, Calabria, Sicilia). Table 2 presents the baseline characteristics of participating children and their parents at the recruitment. There is an equal distribution by sex (51% females). The BMI *z*-score distribution is given in Table 2, signaling that the majority (89.8%) are in the health weight range. Parents’ BMIs are in most cases in a normal range (67.6% of maternal BMIs are normal, 49.5% of paternal BMIs are normal).

Breastfeeding had been started in 92.6% (410/443) of mother–infant pairs participating in the study and it was exclusive in 82.6% (366/443). The rate of breastfeeding at the age of 180 days was 55.5% (246/443). Among children aged ≥12 months (*n* = 377), the rate of breastfeeding at 12 months was 20.2% (76/337).

### 3.2. Daily Dietary Nutrient Intake in the Different Age Groups, Compared with Italian Reference Values 

#### 3.2.1. Daily Dietary Intake of Energy and Macronutrients

Table 3, Table 4, Table 5 and Table 6 report the estimated daily dietary intake of energy and macronutrients in the following age groups: 6 ≤ months < 12 (*n* = 66), 1 ≤ years < 4 (*n* = 148), 4 ≤ years < 7 (*n* = 134), and 7 ≤ years < 11 (95). We reported the mean (SD), median (25th–75th centile), and minimum and maximum values of energy intake (expressed as kcal, kJ, and kcal/kg weight), protein intake (expressed as total grams, % of energy (%E), and g/kg weight), carbohydrate and fat intake (expressed as total grams and %E), and fiber intake (expressed as g/1000 kcal) found in our population. The Italian Reference Values reported are the average requirement (AR), adequate intake (AI), reference intake (RI), population reference intake (PRI), or suggested dietary target (SDT), as applicable [16].

Median daily energy intake increased gradually in accordance with increasing age, and was within the AR reference values range [16] in the 1 ≤ years < 4 and 4 ≤ years < 7 age groups, whereas it was above the AR range in the 6 ≤ months < 12 age group and below the AR range in the 7 ≤ years < 11 group [16].

The median intake of protein, expressed as grams per kilogram body weight per day, reached a maximum of 3.4 g/kg weight in the 1 ≤ years < 4 age group, and decreased to 2.0 g/kg weight in the 7 ≤ years < 11 group. In each age group, the protein intake exceeded the AR reference value for that age group [16]. Regarding the median protein intake expressed as %E, the highest percentage was found in the 1 ≤ years < 4 group (15.9%), and it was outside the RI range (8–12%) [16]. In the age groups 4 ≤ years < 7 and 7 ≤ years < 11, the median %E of protein intake was within the RI range (12–18% [16]) (15.8% and 15.2%, respectively), as was the case for the 6 ≤ months < 12 age group (11.3%, RI 8–12%) [16].

The median intake of carbohydrate as %E increased progressively through age groups, from 48.8% in the 1 ≤ years < 4 age group up to 52.7% in the oldest age group, always remaining within the RI range [16]. Regarding the total amount of carbohydrates, the highest median percentage of simple sugars (25%) was found in the 1 ≤ years < 4 age group, and it decreased to 17.9% in the 7 ≤ years < 11 group; however, the intake of simple sugars exceeded the SDT value (15%) in each age group [16].

Fats were the main component of the diet in the first months of life, and then the percentage reduced, with a median intake of fat as %E below the AI range (40%) [16] and lower than the RI range (35–40%) [16] in the 6 ≤ months < 12 and the 1 ≤ years < 4 age groups, respectively. In the >4-year-old children, the RI reduced to 20–35% and fat %E intake was within the range in both the 4 ≤ years < 7 and 7 ≤ years <11 age groups [16]. However, in all the age groups >6 months, in terms of the total amount of fat, the saturated fatty acid median intake, as %E, was above the SDT (10%) [16] and the polyunsaturated median intake, as %E, was lower than the RI [16]. 

The adequate intake (AI) of fiber, expressed as g/1000 kcal, should be 8.4 g/1000 kcal in each age group [16]; in our population, the median intake of fiber increased progressively, only reaching the AI value in the oldest age group [16].

Table 7 reports, for each age group as applicable, the number of children who exhibited a daily intake of macronutrients outside the RI range and/or above the PRI and/or the SDT value. The highest number of children was within the 1 ≤ years < 4 age group. In this age group, 80 of 148 children (54%) presented a protein intake, as %E, higher than 12% (the upper RI) [16]; 50% of infants aged 12–24 months had a protein intake ≥15%. This is in accordance with the fact that only in the 1 ≤ years < 4 age group was the median intake of protein outside the RI range (8–12%) [16]. In the 6 ≤ months < 12 age group, 34% of toddlers had a protein intake, as %E, higher than 12% (the upper RI) [16]. In agreement with previous results about the excess of protein intake expressed as g/kg weight compared to AR, we have the feedback that 95% of children in the 6 ≤ months < 12 and 7 ≤ years < 11 age groups, and 100% of children in the 1 ≤ years < 4 and 4 ≤ years < 7 age groups, exceeded the PRI [16]. As previously reported, the majority of children exhibited simple sugars and saturated fat intakes ≥SDT, while the polyunsaturated fat consumption was lower than the RI in all age groups [1]. 

#### 3.2.2. Daily Dietary Intake of Calcium, Iron, Zinc, Sodium, and Vitamin D 

Table 8, Table 9, Table 10 and Table 11 report the estimated daily dietary intake of minerals and vitamin D, respectively, in the following age groups: 6 ≤ months < 12 (*n* = 66), 1 ≤ years < 4 (*n* = 148), 4 ≤ years < 7 (*n* = 134), and 7 ≤ years < 11 (95). We reported the mean (SD), median (25th–75th centile), and the minimum and maximum values of the intake of calcium, iron, zinc, and sodium (as mg/day), and of vitamin D (as mcg/day), of our population. We report the Italian Reference Values as applicable: AR, AI, PRI, SDT, and upper level (UL) [16]. 

The median intake of calcium was lower than AR only in the oldest age groups (4 ≤ years < 7 and 7 ≤ years < 11) [16], while in the 6 ≤ months < 12 and 1 ≤ years < 4 age groups, it was above it [16]. Iron, zinc, and sodium intake increased gradually in accordance with increasing age. Iron intake was below the AR in toddlers aged 6–12 months [16], exceeding the reference value in the following age groups [16], while the median intake of zinc and sodium was over the AR/AI reference values [16] (except for zinc intake in 7 ≤ years < 11 children, where it was lower than AR) [16]. The median vitamin D intake was much lower than AR (10 mcg/day) in all the age groups [16].

Table 12 reports, for each age group as applicable, the number of children who exhibited a daily intake of minerals and/or vitamin D above the reference PRI, SDT, and/or UL. A total of 50% of children aged 1 ≤ years < 4 exhibited an intake of calcium >PRI [16], which was not detected in the other age groups; in the same age range, there was the greatest number of children with an iron intake >PRI [16]. Zinc appears the mineral consumed most, exceeding PRI in a consistent number of children >6 months, and in some cases the UL also [16]. In infants aged >12 months, 37% in the 1 ≤ years < 4 age group, 39% in the 4 ≤ years < 7 age group, and 20% in the 7 ≤ years < 11 group had an intake of sodium ≥ SDT [16]. Only eight infants exceeded vitamin D PRI in the 1 ≤ years < 4 age group [16].

## 4. Discussion

### 4.1. Energy and Macronutrients

The results about protein consumption exhibit a median protein intake, expressed as g/kg weight, above the AR in all age of groups [16]; the highest median amount of protein was found during the 1 ≤ years < 4 age range (3.4 g/kg), where 100% of children exceeded PRI (1.0 g/kg) [16]. In the same age, in terms of %E, the majority of children (54%) had a protein intake outside the RI range (8–12%) [16], with a protein consumption ≥ 15% in the 50% sample aged 12–24 months [16]. In the 6 ≤ months < 12 age group, even though the median intake of protein as %E was within the range, 34% of toddlers had a protein intake higher than the upper limit (12%) [16] and 95% of children exceeded the PRI (1.32 g/kg) [16], with a median intake of 2.6 g/kg. Our findings are similar to those raised by the only other national study performed on Italian infants, which reported that the median protein intake increased from 1.2 to 3.3 g/kg from 6 to 36 months of age, respectively, with 84% of children at 9 months and 100% from 12 to 36 months exceeding the PRI for protein [12]. An excess of protein during the first two years of life has been observed in a large number of European studies [18] and protein intake was at or above the upper Italian Dietary Reference Values for European infants [16,17,18]. The European study Childhood Obesity Project (CHOP) reported a total protein intake >15% in more than 50% of European children aged 12 to 24 months [18] and Italy has been found to be one of the European countries with the highest protein intake, together with Spain [18]. Dairy products constitute the main component of the diet up to 12 months, and cow’s milk is generally introduced in the second year of life [18]. Although there was a shift to other protein sources, dairy products still had the highest contribution to dietary protein intakes (%E prot), followed by meat and egg [18]. The main reason for the general protein excess in European countries, starting from nine months, seems to be a substitution, during the complementary feeding, of a too high percentage of dairy protein (including cow’s milk infant formula) by other protein sources, especially meat [18], in a larger extent than recommended. Compared to other countries, in Italy, a higher consumption of meat (%E prot) and a lower consumption of dairy, fruits, and vegetables during the first two years of life [18] have been reported. Nevertheless, the high use of whole cow’s milk instead of formula or breastfeeding before 12 months and the low consumption of legumes in Italian children can explain the high %E covered by animal protein and the relatively low protein intake from fruits and vegetables [18].

Protein consumption above 15% in early life is associated with an increased risk of obesity later in life [18,19,20,21]. Hörnel et al. [22] suggested that total protein intake should not exceed 15% E, whereas Agostoni et al. [23] suggested an upper limit of 14%. The “early protein hypothesis” is driven by the observation that European infants fed with formula containing high proteins levels gained more weight during the first year and had a higher BMI and risk of obesity at the age of six years than those fed with a low protein infant formula more similar to breast milk [19]. Early weight gain is probably caused by higher insulin like growth factor 1 (IGF-1) secretion directly linked to protein intake, especially dairy protein [19,20,21,22]. Feeding habits can impact body weight, but more specifically, body composition. Totzauer et al. observed a greater fat mass deposition from two years onward in children fed with a high protein infant formula during the first year of life, with a two-fold risk for excess body fat at six years of age [24]. 

Lowering the protein intake during the first two years of life, in particular through the promotion of breastfeeding and the use of formulas with a lower content of protein if needed, could be one of the main preventive interventions for obesity and body composition in later age [25]. 

In particular, the present study may signal that dietary habits of Italian children still need to be improved, in particular in the age range from six months to two years, focusing on a reduction of the consumption of animal protein such as meat and cow’s milk.

Another study comparing children’s nutritional habits is "Identification and prevention of dietary-and lifestyle- induced health effects in children and infants" (IDEFICS); this study evaluated the adherence to a Mediterranean-like dietary pattern (i.e., rich in cereals, vegetables, fruit and nuts, and fish and low in meat and dairy products) in young European children and the association with overweight or obesity indicators [26]. It seems that a Mediterranean-like diet is not so common in Mediterranean children, with paradoxically high adherence levels in northern countries in comparison with Mediterranean countries [26]. A “westernized” diet and lifestyle is increasingly taking place in Mediterranean countries, with the highest overweight and obesity prevalence found [26,27]. A Mediterranean dietary pattern seems to be inversely associated with overweight and obesity. The promotion of our original dietary habits appears to be one of the main obesity prevention strategies [26,27].

Regarding carbohydrate consumption, most of the children showed an intake of carbohydrates between 45% and 60% (RI) and this percentage increased with age. Carbohydrates are a good source of vitamins and minerals and they are generally considered healthy and satiating [28]. Simple sugars content consists of both natural sugars (fructose and lactose) and free or added sugars [28]. In the present study, there is a disproportionately low fiber intake from carbohydrates in relation to the amount of simple sugars consumed, particularly in infants aged between six months and four years. Indeed, the median intake of fiber increased progressively, from 6.2 g/1000 kcal in the 6 ≤ months < 12 age group to 8.4 g/1000 kcal in children aged 7 ≤ years < 11, only reaching the AI in the highest age group. The highest median percentage of simple sugars (25%) was found in the 1 ≤ years < 4 age group and decreased up to 17.9% in the group 7 ≤ years < 11. The reduction of simple sugar intake simultaneous to the increase of carbohydrates and fiber intake through age groups is in line with a European study [28], in which the overall decline was mainly accounted for by the reduction in the sugar intake from milk and dairy products [28]. 

However, in the present population the median intake of simple sugars exceeded the SDT value (15% including sugar from whole fruit, milk, and dairy products) at all ages, in line with the only other Italian data that reported very few children at all ages showing an intake of simple sugars lower than the Italian Dietary Reference Values [16]. Free sugars are simple sugars defined by the European Society for Pediatric Gastroenterology, Hepatology and Nutrition (ESPGHAN) as “all monosaccharides/disaccharides added to foods/beverages by the manufacturer/cook/consumer, plus sugars naturally present in honey/syrups/unsweetened fruit juices and fruit juice concentrate” [28]. There is no nutritional requirement for free sugars [29]; they have a high energy density and lead to short-term satiation as “empty calories” [28]. An increase in the frequency of their consumption, resulting in a poor nutritional supply and reduced dietary diversity, can cause an energy imbalance, with an effect on body weight [25,26]. The WHO recommends reducing the intake of free sugars to less than 5% of total daily energy because of the impact of free sugars on body fat deposition, overweight/obesity, and cardiovascular risk, and especially dental caries [28,29]. The consumption of free sugars, particularly sugar sweetened beverages (SSBs), in European children and adolescents exceeds these recommendations. The recent ESPGHAN position paper recommends an intake of free sugars <5% as a desirable goal in children and adolescents aged 2 to 18 years and even less in infants and toddlers <2 years [29]. A healthier approach to beverage and dietary consumption of added sugars should be promoted from infancy, with the aim of preventing negative health effects in later childhood and adulthood [29]. 

The majority of Italian children in this study exhibited a saturated fat intake ≥SDT (10%) [16], according to a previous Italian study [12], with a polyunsaturated fat consumption lower than RI in all age groups (5–10%) [16]. These findings contribute further to an unhealthy dietary pattern. The European CHOP study identified a similar dietary pattern characterized by added sugars, unhealthy fats, and a poor consumption of fish and olive oil as the most common during childhood [30] among European countries. A recent systematic review concluded that dietary patterns that are high in energy-dense, high-fat, and low-fiber foods predispose young people to later overweightness and obesity. Those findings indicate that early interventions to encourage healthy eating habits are imperative.

### 4.2. Minerals and Vitamin D

Several studies reported an important inadequacy regarding the intake of several micronutrients in European children, most frequently vitamin D, calcium, iron, and zinc, since Europe is considered to be a developed region in which nutrition is assumed to be adequate to satisfy population requirements [29]. In our study, Italian children lacked an adequate consumption of calcium starting from the age of four years, confirming that the supposed reduction of dairy intake related to sugar intake declined with increasing age. In the 1 ≤ years < 4 age group, 54% of Italian children had a calcium intake above PRI [16]. The European cohort reported a mild risk for an inadequate intake of calcium, with a prevalence of adequate adherence to recommendations that decreased with increasing age, with the risk of poor bone mineralization from three years onwards when the calcium-rich sources intake is highly replaced with other food groups [31]. 

The median intake of vitamin D is much lower than AR (10 mcg/day) [16] in all the age groups, confirming that it is the most poorly consumed micronutrient, with only a 1–5% prevalence of adequacy reported in European children [31]. This is in line with a broad prevalence of biochemical vitamin D deficiency, which should be considered as an emergent major health problem worldwide. Hence, supplementation of milk and dairy products should be promoted to improve the naturally low content of vitamin D taken through the diet, since skin exposure to UVB radiation, the main source of vitamin D for humans, has been shown to be insufficient [31]. 

Median iron consumption is inadequate in the first year of life in Italian children (below AR) [16]; starting from 12 months onward, the median intake is above the AR, considering the high consumption of meat reported in Italian children [18]. However, if we consider PRI as a cut-point, very few children had a satisfactory iron intake and the result is a general iron deficiency, as reported by previous Italian findings [12], which has to be considered as the common cause of anemia in infants and toddlers [12]. 

Median sodium intake increased gradually; 20–39% of children aged >12 months consumed more sodium than the daily amount recommended as SDT [16]. The majority of European children consume an excessive sodium intake [27]. Sodium intake should be limited during childhood because it is a possible risk factor for the development of cardiovascular disease.

Finally, zinc deficiency is uncommon in Europe [31], but a minimal insufficiency is likely to be more prevalent, with a prevalence of adequacy between 50% and 80% [31]. Our analysis found an adequate median zinc intake above AR [16], except for the 7 ≤ years < 11 age range, where the AR increases to 7 [16]; the percentage of children who had a zinc intake above PRI ranged from 64% in the 1 ≤ years < 4 age group to 15% in the oldest age group [16]. To our knowledge, a zinc deficiency could be associated with immune system dysfunction and delayed physical development and we want to underline that this micronutrient is not of secondary importance [31]. 

## 5. Limitations of the Study

A first limitation of the present study was that it only included Caucasian children. However, it should be noted that, given the current lack of national data in Italy, we were firstly interested in investigating Italian children. Accordingly, not including non-Caucasian families allowed us to narrow down any potential intrinsic underlying differences in eating attitudes among races, also removing possible race biases in estimating the adherence rate to the Italian Nutrients and Energy Reference Intake Levels. A second limitation is that each family used their own food scale. No standardization of instruments was performed. However, parents received instructions and were educated by family pediatricians on the correct method of weighing food. Another limitation of this study is the use self-reported dietary data; however, parents received instructions and were educated by family pediatricians on the correct method of weighing food.

## 6. Conclusions

This is one of the few studies reporting the nutrients intake of Italian children referring to nutrition recommendations in order to identify the principal nutritional errors, both in terms of the excess of some macronutrients and micronutrient deficiencies. An excess of protein consumption has been found in all age of groups: 99% of children (440/443) exceeded the age-related PRI value, with the highest median amount of protein in the 1 ≤ years < 4 age range (3.4 g/kg) [16]. In the same age, in terms of %E, the majority of children (54%) had a protein intake outside the RI range (8–12%) [16]. The majority of the children consumed a quantity of simple carbohydrates (82.3% of the children) and saturated fats (69% of the children) above the Italian Dietary Reference Values. At the same time this study revealed a low intake of fiber and polyunsaturated fats. With regards to median mineral intake of Italian children, vitamin D intake was very low in all age groups. The present results underline the need for an adequate nutritional educative program and healthcare policies starting from the first years of life to ameliorate nutrient intake during childhood, possibly impacting long-term health outcomes.

## Figures and Tables

**Table 1:** Distribution of participating family pediatricians (*n* = 80) and children (*n* = 443) by regional area.

Regional Area ^a^	Family Pediatricians (*n* = 80)	Infants/Children (*n* = 443)
Area 1	17 (21.2)	186 (42)
Area 2	7 (8.7)	16 (3.6)
Area 3	14 (17.5)	51 (11.5)
Area 4	42 (52.5)	190 (42.9)

Data expressed as number of observations (percentage calculated based on the number of valid observations). ^a^ According to Nielsen Italia (Nielsen Italia, Assago, Italy). Area 1: Piemonte, Val d’Aosta, Liguria, Lombardia; Area 2: Trentino-Alto Adige, Veneto, Friuli-Venezia Giulia, Emilia-Romagna; Area 3: Toscana, Umbria, Marche, Lazio, Sardegna (since January 1, 2006); Area 4: Abruzzo, Molise, Puglia, Campania, Basilicata, Calabria, Sicilia.

**Table 2:** Descriptive characteristics of participating children and their parents at recruitment (*n* = 443). Number of valid observations within round brackets.

Characteristic	
***Infant/child***	
Sex (girls) (443)	227 (51.2)
Age (months) (443)	6.4–131
6 ≤ months < 12	66 (14.8)
12 ≤ months < 24	47 (10.6)
24 ≤ months < 36	53 (11.9)
3 ≤ years < 4	48 (10.8)
4 ≤ years < 7	134 (30.2)
7 ≤ years < 11	95 (21.4)
BMI *z*-score (443)	
−2 to <−1	8 (1.8)
−1 to 1	398 (89.8)
>1 to 2	31 (6.9)
>2	7 (1.6)
Breastfeeding	
Started at birth (443)	410 (92.6)
At 6 months (443)	246 (55.5)
At 12 months (377)	20.2 (76)
***Parents***	
Maternal age (year) (416)	36.4 (5.2); 37.2 (33.0–40.0); 24–49
Maternal weight (kg) (408)	63.0 (13.0); 59.9 (55.0–69.0); 43–161
Maternal height (cm) (413)	163.5 (5.8); 164.0 (161.0–168.0); 145.0–181.0
Maternal BMI (kg/m^2^) ^a^ (407)	23.3 (3.9); 22.6 (20.8–25.3); 16.6–40.9
Underweight (<18.5)	20 (4.9)
Health weight range (18.5–25)	275 (67.6)
Pre-obese (>25)	79 (19.3)
Obese (>30)	33 (8.0)
Paternal age (years) (409)	39.2 (5.9); 39.1 (35.0–43.0); 26–79
Paternal weight (kg) (390)	79.9 (11.5); 79.5 (70.0–87.0); 55.0–135.0
Paternal height (cm) (403)	176.2 (6.3); 175.0 (171.0–180.0); 161.0–194.0
Paternal BMI (kg/m^2^) ^a^ (390)	25.7 (3.1); 25.1 (23.5–27.3); 19.4–37.4
Underweight (<18.5)	0 (0.0)
Health weight range (18.5-25)	193 (49.5)
Pre-obese (>25)	160 (41.0)
Obese (>30)	37 (9.5)

Data expressed as mean (SD), median (25th–75th centile), minimum–maximum, or number of observations (percentage calculated based on the number of valid observations). Percentages may not sum to 100 due to rounding. ^a^ BMI, body mass index. Categories adapted from the current WHO classification of adult underweight, overweight, and obesity (see, e.g., [14]).

**Table 3:** Daily dietary intake of energy and macronutrients in infants aged 6 ≤ months < 12 (*n* = 66).

Variable	Mean (SD)	Median (25th–75th Centile)	Min–Max	Reference Values ^a^
Energy				
kcal	803.1 (161.5)	783.0 (692.5–921.0)	488–1304.0	Boys: 620–760 kcal (AR) Girls: 570–690 kcal (AR) depending on age
kJ	3360.3 (675.9)	3276.1 (2897.4–3853.5)	2041.8–5455.9	---
kcal/kg weight	94.7 (29.9)	88.4 (78.4–103.0)	50.9–252.7	---
Protein				
g	23.3 (7.0)	22.7 (18.9–26.3)	9.8–46.3	9 (AR); 11 (PRI)
% Energy	11.5 (2.5)	11.3 (9.7–11.9)	7.3–19.8	8-12 (RI), <15 (VNN)
g/kg weight	2.7 (1.1)	2.6 (2.2–3.1)	1.2–7.9	1.11 (AR); 1.32 (PRI)
Carbohydrate				
g	101.4 (27.6)	94.7 (84.6–116.7)	56.3–194.4	---
% Energy	47.1 (6.3)	46.7 (42–52)	36–61	45-60 (RI)
Simple sugars				
g	51.5 (14.7)	47.6 (39.4–64.1)	30.8–83.8	---
% Energy	24.1 (5.2)	23.4 (19.9–28.6)	13.6–38.0	<15 (SDT)
Fat				
g	33.6 (7.8)	34.3 (26.9–40.0)	18.9–54.5	---
% Energy	37.9 (6.5)	37.3 (32.9–43.0)	24.9–52.1	40 (AI)
Saturated				
g	11.5 (3.0)	11.3 (9.3–13.4)	3.6–19.6	---
% Energy	12.9 (2.4)	13.0 (11.4–14.5)	4.9–17.4	<10 (SDT)
Monounsaturated				
g	15.4(5.0)	15.0 (11.7–18.9)	3.7–27.2	---
% Energy	17.3 (5.0)	16.9 (14.3–21.5)	5.0–30.6	---
Polyunsaturated				
g	3.3 (1.1)	3.3 (2.7–3.7)	0.6–6.7	---
% Energy	3.7 (1.0)	3.7 (3.1–4.1)	0.8–8.2	5-10 (RI)
Fiber				
g	5.2 (2.0)	5.1 (3.4–6.7)	1.9–10.5	---
g/1000 kcal	6.5 (2.3)	6.2 (4.5–8.3)	2.8–12.4	8.4 (AI)

AI, adequate intake, AR; average requirement; PRI, population reference intake; RI, reference intake; SDT, suggested dietary target; VNN, value not explicitly named in [16]. ^a^ According to [11]. --- = No reference values are defined in [16].

**Table 4:** Daily dietary intake of energy, macronutrients, and fiber, in infants/children aged 1 ≤ years < 4 (*n* = 148).

Variable	Mean (SD)	Median (25th–75th Centile)	Min–Max	Reference Values ^a^
Energy				
kcal	1073.4 (217.1)	1064.0 (920.7–1211.0)	628.0–1870.0	Boys:840–1490 kcal (AR) Girls: 770–1370 kcal (AR) depending on age
kJ	4490.9 (908.4)	4451.8 (3852.4–5066.8)	2627.5–7824.0	---
kcal/kg weight	87.5 (23.0)	85.0 (73.3–101.3)	18.1–158.4	---
Protein				
g	42.9 (11.5)	42.4 (36.0–49.0)	17.1–78.5	11 (AR); 14 (PRI); infants:
% Energy	16.0 (3.0)	15.9 (14.2–18.1)	7.8–25.6	8–12 (RI) and <15 (VNN); otherwise 12–18 (RI)
g/kg weight	3.5 (1.1)	3.4 (2.7–4.1)	1.4–8.7	0.82 (AR); 1.0 (PRI)
Carbohydrate				
g	139.7 (35.7)	138.6 (116.5–160.6)	11.7–317.5	---
% Energy	49.0 (6.1)	48.8 (45.6–53.0)	30.5–63.7	45–60 (RI)
Simple sugars				
g	57.2 (17.9)	58.5 (44.1–67.6)	22.8–113.1	---
% Energy	28.6 (12.2)	25.0 (20.3–37.0)	9.8–62.6	<15 (STD)
Fat				
g	40.4 (11.1)	39.2 (32.7–46.7)	15.8–83.5	---
% Energy	33.7 (5.7)	33.8 (30.0–36.7)	22.0–57.3	35–40 (RI)
Saturated				
g	14.4 (5.1)	13.8 (10.8–17.1)	4.4–30.4	---
% Energy	12.0 (3.1)	12.1 (10.0–13.7)	5.1–21.3	<10 (STD)
Monounsaturated				
g	15.2(5.6)	14.8 (11.9–18.3)	4.3–49.4	---
% Energy	12.6 (4.0)	10.0 (12.5–14.8)	4.1–33.9	---
Polyunsaturated				
g	3.5 (1.2)	3.3 (2.5–4.3)	1.1–7.0	---
% Energy	2.9 (0.9)	2.8 (2.2–3.4)	1.2–6.0	5–10 (RI)
Fiber				
g	8.4 (3.0)	8.1 (6.3–10.6)	3.2–15.6	---
g/1000 kcal	7.9 (2.5)	7.7 (5.9–9.6)	2.5–16.2	8.4 (AI)

AI, adequate intake; AR, average requirement; PRI, population reference intake; RI, reference intake; SDT, suggested dietary target; VNN, value not explicitly named in [16]. ^a^ According to [11]. --- = No reference values are defined in [16].

**Table 5:** Daily dietary intake of energy, macronutrients, and fiber, in children aged 4 ≤ years < 7 (*n* = 134).

Variable	Mean (SD)	Median	Min–Max	Reference Values ^a^
		(25th–75th Centile)		
Energy				
kcal	1331.2 (300.0)	1294.0 (1135.5–1473.5)	546.0–2289.0	Boys: 1330–1880 kcal (AR) Girls: 1220–1740 kcal (AR) depending on age
kJ	5569.9 (1255.0)	5414.1 (4750.9–6165.1)	2284–9544.2	---
kcal/kg weight	70.3 (18.6)	68.4 (59.2–79.5)	29.3–136.4	---
Protein				
g	51.6 (13.2)	49.0 (42.6–58.9)	21.3–104.2	16 (AR); 19 (PRI)
% Energy	15.6 (2.6)	15.2 (13.8–17.1)	10.4–27.0	12–18 (RI)
g/kg weight	2.7 (0.8)	2.5 (2.2–3.1)	1.0–6.0	0.76 (AR); 0.94 (PRI)
Carbohydrate				
g	185.6 (48.4)	181.4 (150.8–213.4)	76.2–323.0	---
% Energy	52.2 (5.9)	52.3 (48.0–56.0)	38.7–68.1	45–60 (RI)
Simple sugars				
g	66.9 (26.2)	65.1 (48.7–80.9)	9.9–155.3	---
% Energy	18.6 (5.6)	18.3 (14.7–21.7)	6.8–34.4	<15 (SDT)
Fat				
g	47.1 (13.3)	45.8 (37.9–53.8)	9.7–108.7	---
% Energy	31.8 (5.1)	31.6 (27.9–35.3)	15.9–43.3	20–35 (RI)
Saturated				
g	17.1 (5.1)	16.5 (13.3–21.2)	1.8–30.5	---
% Energy	11.4 (2.7)	11.3 (9.6–13.2)	3.0–18.3	<10 (STD)
Monounsaturated				
g	17.2 (5.9)	16.0 (13.0–20.2)	4.9–41.4	---
% Energy	11.6 (2.8)	11.2 (9.5–13.9)	7.0–18.3	---
Polyunsaturated				
g	4.9 (2.5)	4.4 (3.7–5.5)	1.8–25.4	---
% Energy	3.3 (1.1)	3.0 (2.6–3.8)	1.5–10.0	5-10 (RI)
Fiber				
g	11.1 (4.3)	10.3 (8.4–13.4)	2.4–29.6	---
g/1000 kcal	8.3 (2.7)	8.1 (6.4–9.6)	2.9–19.3	8.4 (AI)

AI, adequate intake; AR, average requirement; PRI, population reference intake; RI, reference intake; SDT, suggested dietary target. ^a^ According to [16]. --- = No reference values are defined in [15].

**Table 6:** Daily dietary intake of energy, macronutrients, and fiber, in children aged 7 ≤ years < 11 (*n* = 95).

	Mean (SD)	Median	Min–Max	Reference Values ^a^
		(25th–75th Centile)		
Energy				
kcal	1424.4 (317.7)	1425.0 (1220.0–1615.0)	604.0–2414.0	Boys: 1580–2460 kcal (AR)
				Girls: 1470–2230 kcal (AR)
				depending on age
kJ	5959.7 (1329.4)	5962.2 (5104.5–6757.2)	2527.1–10100.0	---
kcal/kg weight	54.5 (16.8)	52.2 (41.2–67.4)	21.3–103.2	---
Protein				
g	54.7 (13.9)	54.1 (45.3–61.1)	29.9–107.6	25 (AR); 31 (PRI)
% Energy	15.5 (2.6)	15.2 (13.6–17.2)	10.6–26.8	12-18 (RI)
g/kg weight	2.1 (0.6)	2.0 (1.6–2.5)	1.0–3.8	0.81 (AR); 0.99 (PRI)
Carbohydrate				
g	199.1 (53.4)	200.3 (153.8–231.5)	55.9–365.2	---
% Energy	52.2(6.2)	52.7 (48.0–56.4)	34.7–69.8	45-60 (RI)
Simple sugars				
g	66.2 (20.6)	64.8 (52.3–78.9)	27.8–127.2	---
% Energy	17.7 (4.6)	17.9 (14.2–20.7)	7.3–28.1	<15 (SDT)
Fat				
g	50.5 (13.4)	50.0 (42.3–59.2)	22.7–90.7	---
% Energy	32.0 (5.1)	32.0 (28.5–35.0)	18.3–45.4	20–35 (RI)
Saturated				
g	17.0 (5.2)	16.1 (12.9–20.0)	7.7–32.4	---
% Energy	10.8 (2.5)	10.5 (9.0–12.4)	6.2–19.0	<10 (SDT)
Monounsaturated				
g	19.3(5.9)	19.1 (16.0–22.8)	7.1–41.3	---
% Energy	12.3 (3.0)	12.1 (9.9–14.5)	6.5–19.7	---
Polyunsaturated				
g	4.9 (1.3)	4.8 (3.9–5.8)	1.5–8.7	---
% Energy	3.2 (0.7)	3.2 (2.6–3.6)	1.7–5.0	5-10 (RI)
Fiber				
g	12.0 (3.6)	11.7 (10.1–14.0)	2.9–22.7	---
g/1000 kcal	8.4 (2.0)	8.4 (7.1–9.4)	3.9–14.7	8.4 (AI)

AI, adequate intake; AR, average requirement; PRI, population reference intake; RI, reference intake; SDT, suggested dietary target. ^a^ According to [16]. --- = No reference values are defined in [16].

**Table 7:** Number of infants/children who exhibited a daily intake of macronutrients outside the reference RI range and/or above the reference PRI and/or STD value, by age.

Variable	Reference ^a^	Age			
		6 < months < 12	1 < years < 4 (*n* = 148)	4 < years < 7	7 < years < 11
		(*n* = 66)		(*n* = 134)	(*n* = 95)
Protein					
% Energy	outside RI	25	82	32	18
	<lower RI	2	2	5	6
	>upper RI	23	80	27	12
	≥15 (VΝΝ)	6	35 ^b^	na	na
g	> PRI	64	148	134	94
g/kg weight	> PRI	63	148	134	91
Carbohydrate					
% Energy	outside RI	13	37	32	16
	<lower RI	12	31	21	11
	>upper RI	1	6	11	5
Simple sugars					
%Energy	≥ ΣΤΔ	64	134	99	68
Fat					
%Energy	outside RI	na	102	37	25
	<lower RI		88	1	1
	>upper RI		14	36	24
Saturated					
%Energy	≥ ΣΤΔ	58	111	83	54
Monounsaturated					
%Energy	---	na	na	na	na
Polyunsaturated					
%Energy	outside RI	62	141	122	94
	<lower RI	62	141	122	94
	>upper RI	0	0	0	0

PRI, population reference intake; RI, reference intake; SDT, suggested dietary target; VNN, value not explicitly named in [16]. na, not applicable. ^a^ According to [16]. --- = No reference values are defined in [16] ^b^ In infants aged 12–24 months (*n* = 70); not applicable in children aged 2 < years < 4 (*n* = 78). Missing data. Age 6 ≤ months < 12: saturated, monounsaturated and polyunsaturated fats (*n* = 1), and fiber (*n* = 1). Age 1 ≤ years < 4: simple sugars (*n* = 2), and saturated, monounsaturated, and polyunsaturated fats (*n* = 2). Age 4 ≤ years < 7: sugar (*n* = 1); saturated, monounsaturated, and polyunsaturated fats (*n* = 1), and fiber (*n* = 1). Age 7 ≤ years < 11: protein, expressed as g/kg weight, (*n* = 1).

**Table 8:** Daily dietary intake of minerals and vitamin D in infants aged 6 ≤ months < 12 months (*n* = 66) ^┼^.

Variable	Mean (SD)	Median (25th–75th Centile)	Min–Max	Reference Values ^a^
Calcium (mg/day)	513.7 (193.5)	465.9 (356.6–647.1)	196.1–1019.9	260 (AI)
Iron (mg/day)	5.3 (3.0)	4.9 (2.8–7.3)	1.0–14.4	7 (AR); 11 (PRI)
Zinc (mg/day)	3.5 (1.2)	3.5 (2.7–4.0)	1.3–6.7	2 (AR); 3 (PRI)
Sodium (g/day)	0.55 (0.38)	0.46 (0.29–0.62)	0.09–2.05	0.4 (AI)
Vitamin D (mcg/day)	4.0 (3.5)	3.5 (0.6–7.8)	0.1–10.7	10 (AI); 40 (UL)

AI, adequate intake; AR, average requirement; PRI, population reference intake; UL, tolerable upper intake level. ^a^ According to [16]. ^┼^ As calculated from ingested food. No data were available about the intake of minerals and vitamins from other sources, such as supplements, medicines, or others.

**Table 9:** Daily dietary intake of minerals and vitamin D in infants/children aged 1 ≤ years < 4 (*n* = 148) ^┼^.

Variable	Mean (SD)	Median (25th–75th Centile)	Min–Max	Reference Values ^a^
Calcium (mg/day)	613.1 (237.6)	606.6 (430.8–780.4)	187.2–1277.7	450 (AR); 600 (PRI)
Iron (mg/day)	6.0 (2.6)	5.4 (4.1–7.3)	0.9–15.2	4 (AR); 8 (PRI)
Zinc (mg/day)	5.5 (1.6)	5.5 (4.2–6.4)	2.1–11.5	4 (AR); 5 (PRI); 7 (UL)
Sodium (g/day)	0.84 (0.41)	0.72 (0.56–1.03)	0.21–2.42	0.7 (AI); 0.9 (SDT)
Vitamin D (mcg/day)	3.6 (5.0)	1.3 (0.8–3.8)	0.2–23.9	10 (AR); 15 (PRI); 65 (UL)

AI, adequate intake; AR, average requirement; PRI, population reference intake; UL, tolerable upper intake level. ^a^ According to [16]. ^┼^ As calculated from ingested food. No data were available about the intake of minerals and vitamins from other sources, such as supplements, medicines, or others.

**Table 10:** Daily dietary intake of minerals and vitamin D in children aged 4 ≤ years < 7 (*n* = 134) ^┼^.

Variable	Mean (SD)	Median (25th–75th Centile)	Min–max	Reference Values ^a^
Calcium (mg/day)	495.4 (188.8)	477.5 (379.0–613.8)	133.6–1126.4	700 (AR); 900 (PRI)
Iron (mg/day)	5.7 (2.2)	5.4 (4.1–7.0)	2.2–16.6	5 (AR); 11 (PRI)
Zinc (mg/day)	6.0 (1.8)	5.7 (4.7–7.0)	2.5–12.4	5 (AR); 6 (PRI); 10 (UL)
Sodium (g/day)	1.16 (0.48)	1.09 (0.85–1.41)	0.12–3.6	0.9 (AI); 1.2 (SDT)
Vitamin D (mcg/day)	1.4 (1.4)	1.1 (0.7–1.7)	0.3–10.6	10 (AR); 15(PRI); 75 (UL)

AI, adequate intake; AR, average requirement; PRI, population reference intake; UL, tolerable upper intake level. ^a^ According to [16]. ^┼^ As calculated from ingested food. No data were available about the intake of minerals and vitamins from other sources, such as supplements, medicines, or others.

**Table 11:** Daily dietary intake of minerals and vitamin D in children aged 7 ≤ years < 11 (*n* = 95) ^┼^.

Variable	Mean (SD)	Median (25th–75th Centile)	Min–Max	Reference Values ^a^
Calcium (mg/day)	459.4 (180.5)	433.2(326.6–589.5)	126.2–964.4	900 (AR); 1100 (PRI)
Iron (mg/day)	6.0 (2.1)	5.6 (4.5–7.3)	2.4–16.4	5 (AR); 13 (PRI)
Zinc (mg/day)	6.2 (1.6)	6.1 (4.8–7.3)	2.7–10.8	7 (AR); 8 (PRI); 13 (UL)
Sodium (g/day)	1.21 (0.45)	1.20 (0.91–1.45)	0.24–2.77	1.1 (AI); 1.5 (SDT)
Vitamin D (mcg/day)	1.3 (1.0)	1.0 (0.7–1.4)	0.2–5.7	10 (AR); 15 (PRI); 75(UL)

AI, adequate intake; AR, average requirement; PRI, population reference intake; UL, tolerable upper intake level. ^a^ According to [16]. ^┼^ As calculated from ingested food. No data were available about the intake of minerals and vitamins from other sources, such as supplements, medicines, or others.

**Table 12:** Number of infants/children who exhibited a daily intake of minerals and/or vitamin D above the reference PRI, STD, and/or UL value, by age ^┼^.

Variable	Reference ^a^	Age			
		6 ≤ months < 12 (*n* = 66)	1 ≤ years < 4 (*n* = 148)	4 ≤ years < 7 (*n* = 134)	7 ≤ years < 11 (*n* = 95)
Calcium (mg)	> PRI	Na	75	4	0
Iron (mg)	> PRI	2	29	3	1
Zinc (mg)	> PRI	44	95	61	15
	> UL	Na	20	5	0
Sodium (g)	≥ SDT	Na	56	53	9
Vitamin D (mcg)	> PRI	na	8	0	0
	> UL	0	0	0	0

PRI, population reference intake; SDT, suggested dietary target; UL, tolerable upper intake level. na, not applicable. ^a^ According to [16]. ^┼^ As calculated from ingested food. No data were currently available about the intake of minerals and vitamins from other sources, such as supplements, medicines, or others. Missing data. Age 6 ≤ months < 12: zinc (*n* = 1). Age 1 ≤ years < 4: sodium (*n* = 2). Age 4 ≤ years < 7: sodium (*n* = 1), vitamin D (*n* = 2). Age 7 ≤ years < 11: none.
